# A Novel Algorithm for Analysis of Multiple Endpoints Using Risk–Benefit Profiles

**DOI:** 10.1093/geroni/igab046.2989

**Published:** 2021-12-17

**Authors:** Natalia Gouskova, Dae Kim, Sandra Shi, Thomas Travison

**Affiliations:** 1 Hebrew SeniorLife, Marcus Institute for Aging Research, Hebrew SeniorLife, Boston, Massachusetts, United States; 2 Harvard Medical School, Boston, Massachusetts, United States; 3 Hebrew SeniorLife, Harvard Medical School, Roslindale, Massachusetts, United States

## Abstract

Often it is necessary to evaluate effectiveness of an intervention on the basis of multiple event outcomes of variable benefit and harm, which may develop over time. An attractive approach is to order combinations of these events based on desirability of the overall outcome (e.g. from cure without any adverse events to death), and then determine whether the intervention shifts the distribution of these ordered outcomes towards more desirable (Evans, Follmann 2016). The win ratio introduced in Pocock et al 2012 was an earlier implementation of this approach. More recently Claggett et al 2015 proposed a more comprehensive method allowing nonparametric and regression-based inference in presence of competing risks. Key to the method is weighting observations by inverse probability of censoring (IPC) processes specific to participants and event types. The method has seemingly great practical utility, but computation of weights is a non-trivial challenge with real-life data when each event can have its own censoring time. We present a novel recursive algorithm solving this problem for an arbitrary number of events ordered by clinical importance or desirability. The algorithm can be implemented in SAS or R software, and computes IPC weights, as well as nonparametric or parametric estimates and resampling-based measures of uncertainty. We illustrate the approach using data from the SPRINT trial of antihypertensive intervention, comparing risk-benefit profiles for robust, pre-frail, and frail subpopulations, and in analysis of fall as a function of progressive risk factors. More general use of the software tools deploying the method is described.

